# Limited impact of weekend admissions on hip fracture outcomes in elderly patients: A study from a Japanese nationwide medical claims database

**DOI:** 10.1111/ggi.15041

**Published:** 2024-12-04

**Authors:** Yu Mori, Kunio Tarasawa, Hidetatsu Tanaka, Naoko Mori, Kiyohide Fushimi, Toshimi Aizawa, Kenji Fujimori

**Affiliations:** ^1^ Department of Orthopaedic Surgery Tohoku University Graduate School of Medicine Sendai Japan; ^2^ Department of Health Administration and Policy Tohoku University Graduate School of Medicine Sendai Japan; ^3^ Department of Radiology Akita University Graduate School of Medicine Akita Japan; ^4^ Department of Health Policy and Informatics Tokyo Medical and Dental University Graduate School of Medicine and Dental Sciences Tokyo Japan

**Keywords:** hip fracture, mortality, osteoporosis, sequlae, weekend admission

## Abstract

**Aim:**

The effectiveness of early surgery in preventing complications in elderly Japanese hip fracture patients and the impact of weekend hospitalization need further investigation. The purpose of this study was to determine whether weekend hospitalization affects the incidence of various sequelae and death during hospitalization in elderly hip fracture patients using a comprehensive Japanese hip fracture case database.

**Methods:**

We retrospectively analyzed the Japanese National Administrative DPC (Diagnosis Procedure Combination) database from April 2016 to March 2022. During this period, approximately 1100 DPC‐affiliated hospitals consistently provided medical records with consent for the study. The study focused on weekend hospitalizations and investigated the associations with postoperative pneumonia, pulmonary embolism, myocardial infarction, urinary tract infection, acute renal dysfunction, dementia, and in‐hospital mortality after propensity score matching. Owing to the large population size of the study, significance levels were strictly enforced, and a *P*‐value < 0.001 was considered statistically significant.

**Results:**

After performing propensity score matching based on age, sex, and comorbidities, 111 035 patient pairs were identified, comparing those admitted on weekends versus weekdays. The analysis showed no heightened risk of sequelae for those admitted during the weekend compared with weekdays. Additionally, there was a slight trend toward higher mortality risk during weekend hospital stays; however, the increase was insignificant, with a hazard ratio of 1.071 (95% confidence interval: 1.005–1.140, *P* = 0.03).

**Conclusion:**

The results of this study indicate that weekend hospitalization for elderly patients with hip fractures is not definitively associated with an increase in various sequelae or in‐hospital mortality and that the importance of early surgery for elderly patients with hip fractures may be recognized and promoted in Japan. **Geriatr Gerontol Int 2025; 25: 75–81**.

## Graphical abstract

This study indicates that weekend hospitalization for elderly patients with hip fractures is not definitively associated with an increase in various sequelae or in‐hospital mortality.

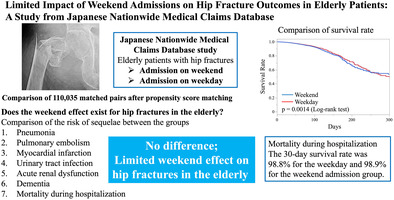

## Introduction

Hip fractures represent a prevalent orthopedic condition in the elderly population and are correlated with considerable morbidity and elevated mortality rates.^1^, ^2^ As the elderly population in the United States increases, the incidence of hip fractures is projected to double, rising from 250 000 cases in 1990 to an estimated 500 000 by 2040.^3^ In Japan, which has an aging population, there are 13 million individuals diagnosed with osteoporosis,^4^ and the number of patients suffering from hip fractures is estimated to be 250 000.^5^ Consequently, osteoporosis‐related hip fractures represent a global issue, affecting populations irrespective of country or region. Over 90% of these fractures occur in individuals aged 65 years and older. Additionally, the prevalence of pre‐injury complications in patients with femoral neck fractures tends to rise over time. The 30‐day mortality rate for these fractures ranges from 4.0% to 5.4%,^6^ and an estimated 1‐year mortality rate of 25% has been reported for hip fractures.^7^

There is an extensive amount of literature that discusses the impact of timely hip fracture surgery on morbidity and mortality rates.^8^, ^9^, ^10^, ^11^ Therefore, surgery within 48 h of hip fracture is recommended. In contrast, the impact of weekend hospitalization for hip fractures is controversial. There are reports that it increases the risk of mortality and complications and, conversely, reports that it does not increase the risk of mortality and complications.

The “weekend effect,” a concept suggesting that outcomes for patients hospitalized for hip fractures over weekends are worse compared with those admitted on weekdays, has been extensively studied within the context of hip fracture treatment. A study from the Norwegian Hip Fracture Register, which included 76 410 cases of hip fractures, reported that patients who sustained a hip fracture over a weekend experienced a slightly higher mortality rate within the first 2 months postoperatively, with a hazard ratio of 1.08 and a 95% confidence interval (CI) of 1.03–1.14. Interestingly, this increased mortality risk did not continue beyond the initial months, though the impact of being admitted over the weekend was still evident at the 1‐year follow‐up.^12^

In contrast, a study conducted in the United States between 1998 and 2010 using the Nationwide Inpatient Sample found that patients admitted on weekends had lower mortality rates and shorter average lengths of stay compared with patients admitted on weekdays.^13^ Similarly, another study concluded that hip fracture patients admitted on weekends do not have an increased risk of 30‐day mortality compared with those admitted on weekdays.^14^ This finding is supported by other studies showing a limited weekend effect in patients hospitalized with hip fractures.^15^, ^16^ On the other hand, while these studies have predominantly focused on Western populations, there have been no large‐scale studies examining the “weekend effect” on hip fracture outcomes among Asian or Japanese populations.

Studies using the Diagnosis Procedure Combination (DPC) database in Japanese hip fracture cases have previously reported the impact of dementia^17^ and the results of a study on the length of hospital stay.^18^ The benefits of early surgery for the prevention of sequelae of hip fractures and the utility of total hip replacement surgery in improving function have also been discussed.^19^, ^20^, ^21^ Furthermore, a relationship between rheumatoid arthritis and postoperative complications such as pneumonia, pulmonary embolism, and in‐hospital mortality has been reported.^22^ On the other hand, the impact of weekend admissions on hip fractures has not yet been studied. The incidence of sequelae may increase owing to surgery for hip fractures being postponed over the weekend, followed by hospitalization. However, with the growing availability of early surgery for hip fractures, emergency procedures can now be carried out on the day of admission or soon after the weekend. We hypothesize that the impact of hip fractures occurring over the weekend and subsequent hospitalization on the development of sequelae might be limited. Therefore, the purpose of this study was to determine whether weekend hospitalization is associated with the occurrence of sequelae such as pneumonia, pulmonary embolism, urinary tract infection, myocardial infection, and mortality due to hospitalization sequelae in elderly patients with hip fractures, using a large database of hip fractures in the Japanese population.

## Methods

### Study design

This retrospective study adhered to the ethical guidelines of the Declaration of Helsinki and received approval from the Tokyo Medical and Dental University (approval number: M2000‐788). Data was retrospectively collected from the Japanese National Administrative DPC reimbursement system database.^23^ At admission, each hospital obtained extensive consent from the patient, which included permissions for the type and course of treatment and the academic use of data gathered during their care. Furthermore, this paper does not include any information that could identify the participants. The period of the study spanned from April 2016 to March 2022. During this time, around 1100 hospitals eligible under the DPC system regularly submitted their medical records and agreed to their use for research purposes. Patients who received treatment for hip fractures at these hospitals across Japan were included in further analyses. This data accurately represents real clinical practice in the country. Clinical research concentrated on proximal femur fractures in the elderly, defined as individuals aged 65 years and older. Hip fractures were identified using the International Statistical Classification of Diseases, Tenth Revision (ICD‐10) codes: S7200 for femoral neck fractures, S7210 for trochanteric fractures, and S7220 for subtrochanteric fractures. For the hip fracture cohorts, patients were chosen from a registry that categorized entries based on three criteria: (i) principal diagnosis, (ii) primary reason for admission, and (iii) condition consuming the most medical resources.

### Propensity score matching

Weekend admissions were defined as admissions on Friday, Saturday, or Sunday. A one‐to‐one propensity score (PS) matching between hip fracture cases with weekday and weekend admissions was performed. Confounding factors adjusted in the analysis were age, sex, and comorbid conditions, including hypertension, dementia, ischemic heart disease, cerebrovascular disease, chronic renal dysfunction, chronic lung disease, and diabetes. The model's discriminatory ability was evaluated using C‐statistics. PS estimates facilitated nearest‐neighbor matching without replacement, utilizing these scores as calipers set at 0.2 times the standard deviation of the PS estimate. This approach generated matched pairs, forming weekday and weekend admission groups based on PS matching.

### Statistical analyses

Data are presented as mean ± standard deviation. The differences between the two groups were analyzed using the χ^2^ test and Student's *t*‐test for each clinical parameter, comparing weekday and weekend admissions. For the variables of sequelae and mortality that showed significant or near‐significant associations with weekend admissions, multivariate logistic regression analysis was conducted to investigate correlations with age, sex, weekend admission, and comorbidities, aiming to identify independent risk factors. The log‐rank test was performed to compare the difference in survival rate between the groups. Owing to the large sample size of the study, strict significance thresholds were established. All statistical tests were two‐tailed, and *P*‐values < 0.001 were deemed statistically significant. Statistical analyses were carried out using JMP, version 17 (SAS, Cary, NC, USA).

## Results

**Figure 1:**
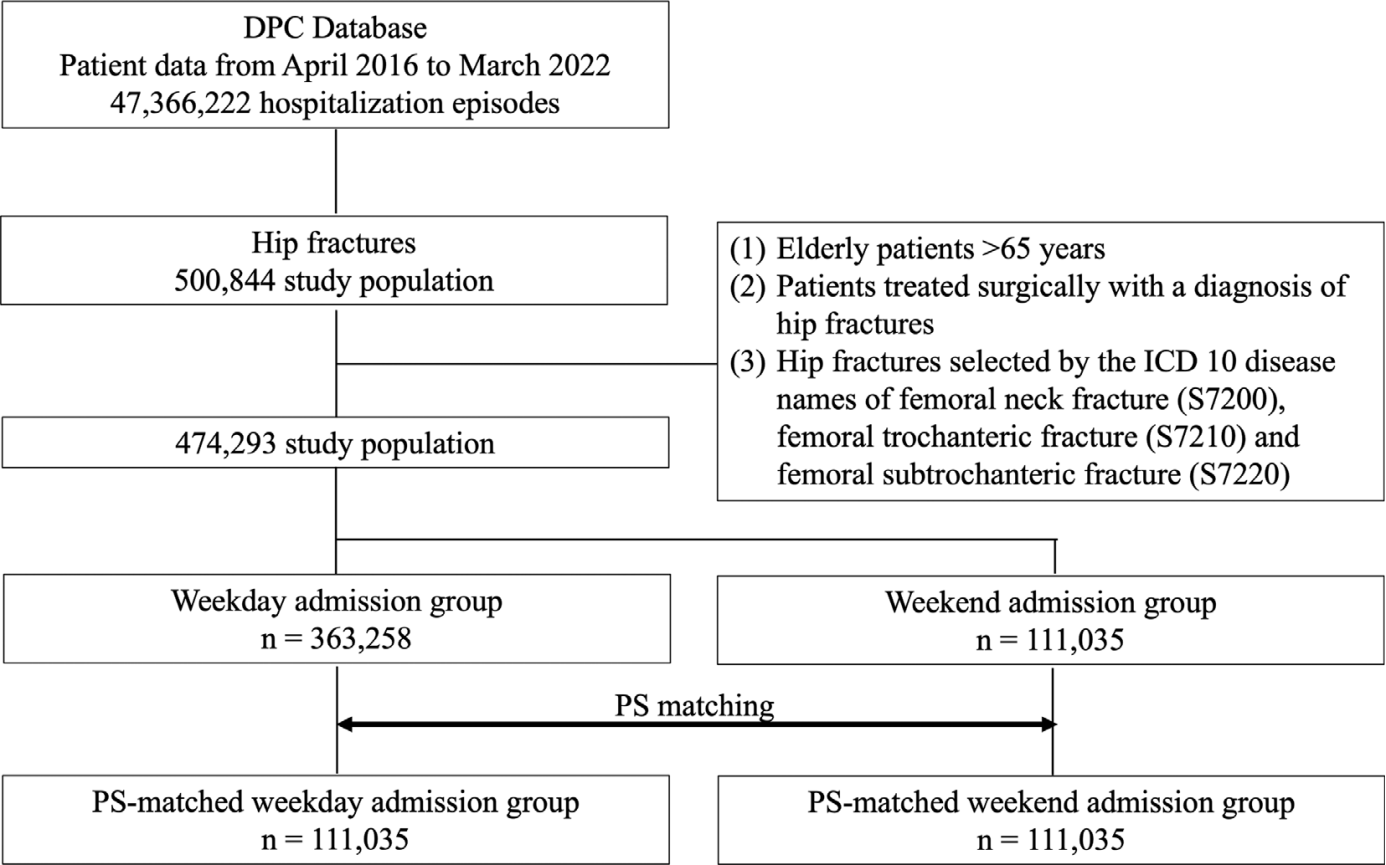
Flow diagram of patient selection for elderly hip fractures and propensity score (PS) matching. This diagram shows how eligible patients were extracted from the DPC (Diagnosis Procedure Combination) database and the PS matching between weekday and weekend admissions patients.

Figure 1 illustrates the schematic model of the patient selection process: a total of 500 844 patients who met the inclusion and exclusion criteria were chosen from patient data spanning from April 2016 to March 2022. Out of these, 111 035 patients were admitted during weekends, and 363 258 were admitted on weekdays. Following one‐to‐one PS matching based on age, sex, and comorbidity, the groups for both weekend and weekday admissions consisted of 111 035 cases each. Table 1 presents the characteristics of patients admitted on weekends and weekdays. Before PS matching, no differences were observed in age, sex, or complication rates among hip fracture patients admitted on weekends compared with on weekdays. Paradoxically, patients admitted on weekends were significantly more likely to undergo surgery within 2 days of admission and had shorter durations of hospital stays. Conversely, the weekend admission group required significantly more blood transfusions post‐surgery. However, there was no significant difference in the initiation of rehabilitation within 3 days of admission between the two groups.

**Table 1:** Characteristics of patients before and after propensity score matching

	Before PS matching			After PS matching		
	Weekday group	Weekend group	*P*‐value	PS‐matched weekday group	PS‐matched weekend group	SMD
*n*	363 258	111 035		111 035	111 035	
Age	84.4 ± 7.7	84.4 ± 7.7	0.025	84.4 ± 7.7	84.4 ± 7.7	0.0006
Sex						
Men	78 855 (21.7%)	24 309 (21.9%)	0.19	24 335 (21.9%)	24 309 (21.9%)	0.0005
Women	284 403 (78.3%)	86 726 (78.1%)		86 700 (78.1%)	86 726 (78.1%)	
Comorbidities						
Hypertension	140 072 (38.6%)	42 740 (38.5%)	0.68	42 663 (38.4%)	42 740 (38.5%)	0.0014
Dementia	80 018 (22.0%)	24 203 (21.8%)	0.1	24 166 (21.8%)	24 203 (21.8%)	0.0007
Diabetes	67 093 (18.5%)	20 774 (18.7%)	0.07	20 755 (18.7%)	20 774 (18.7%)	0.0003
Cerebrovascular disease	37 385 (10.3%)	11 347 (10.2%)	0.49	11 326 (10.2%)	11 347 (10.2%)	0.0003
Ischemic heart disease	29 106 (8.0%)	9060 (8.2%)	0.12	8985 (8.1%)	9060 (8.2%)	0.0022
Chronic renal dysfunction	19 512 (5.4%)	6051 (5.4%)	0.31	6036 (5.4%)	6051 (5.4%)	0.0006
Chronic lung disease	5403 (1.5%)	1707 (1.5%)	0.42	1635 (1.5%)	1707 (1.5%)	0.0019

						χ^2^ statistics	*P*‐value
Fracture type							
Femoral neck	183 794 (50.6%)	54 631 (49.9%)	<0.0001	56 116 (50.5%)	54 631 (49.9%)	42.4	<0.0001
Femoral trochanteric	171 614 (47.2%)	54 026 (48.0%)		52 494 (47.3%)	54 026 (48.0%)		
Femoral subtrochanteric	7850 (2.2%)	2378 (2.1%)		2425 (2.2%)	2378 (2.1%)		
Surgery							
ORIF	226 578 (62.4%)	71 872 (64.8%)	<0.0001	69 336 (62.4%)	71 872 (64.8%)	131.1	<0.0001
BHA	131 645 (36.2%)	37 784 (34.0%)		40 135 (36.2%)	37 784 (34.0%)		
THA	5035 (1.4%)	1379 (1.2%)		1564 (1.4%)	1379 (1.2%)		
Surgery within 2 days of admission	162 176 (44.6%)	64 760 (58.3%)	<0.0001	49 629 (44.7%)	64 760 (58.3%)	4140	<0.0001
Surgery after 2 days of admission	201 082 (55.4%)	46 275 (41.7%)		61 406 (55.3%)	46 275 (41.7%)		
Blood transfusion	0.44 ± 1.06	0.47 ± 1.10	<0.0001	0.44 ± 1.06	0.47 ± 1.10		<0.0001
Rehabilitation							
Rehabilitation within 3 days of admission	177 686 (48.9%)	53 974 (48.6%)	0.076	54 230 (48.8%)	53 974 (48.6%)	1.2	0.28
Rehabilitation after 3 days of admission	185 572 (51.1%)	57 061 (51.4%)		56 805 (51.2%)	57 061 (51.4%)		
Length of hospitalization	35.6 ± 27.6	34.7 ± 29.4	<0.0001	35.6 ± 27.2	34.7 ± 29.4		<0.0001

One‐to‐one PS matching was performed.

Age is shown as mean ± standard deviation; *P*‐values of <0.001 are considered significant by the Student's *t*‐test and χ^2^ test.

BHA, bipolar hemiarthroplasty; ORIF, open reduction and internal fixation; PS, propensity score; SMD, standard mean difference; THA, total hip arthroplasty.

The C statistic was calculated to be 0.725. After PS matching, the standardized mean differences for all measured parameters were less than 0.1, indicating well‐balanced groups. The weekend admission group had a higher incidence of femoral trochanteric fractures, whereas the weekday group primarily experienced femoral neck fractures, with a statistically significant difference (*P* < 0.0001). Furthermore, weekday admissions were more commonly associated with bipolar hemiarthroplasty for femoral neck fractures, whereas weekend admissions had a higher occurrence of osteosynthesis for fractures of the femoral neck, trochanteric, and subtrochanteric regions (*P* < 0.0001). Notably, 58.3% of patients in the weekend group underwent surgery within 2 days of admission, compared with 44.7% in the weekday group. The volume of blood transfusions on the day of surgery was also significantly higher in the weekend group (0.44 ± 1.06 vs. 0.47 ± 1.10, *P* < 0.0001). Approximately 49% of patients received rehabilitation within 3 days of admission, although whether this occurred before or after surgery was not specified. No significant differences in the timing of rehabilitation initiation were observed between the weekend and weekday groups. The average length of hospital stay was significantly shorter for the weekend group, at 34.7 ± 29.4 days, compared with 35.6 ± 27.2 days for the weekday group, with a statistically significant difference (*P* < 0.0001).

Table 2 compares the incidence of sequelae before and after PS matching. There were no differences between the groups in the incidence of secondary pneumonia, pulmonary embolism, myocardial infarction, urinary tract infection, acute renal failure, or dementia. In contrast, there was a trend toward a higher incidence of mortality during hospitalization, although not significant, in the weekend hospitalization group after PS matching (1929 [1.7%] vs. 2061 [1.9%], *P* = 0.035).

**Table 2:** Comparison of sequelae before and after propensity score matching

	Before PS matching			*P*‐value	After PS matching			*P*‐value
	Weekday group	Weekend group	χ^2^ statistics		PS‐matched weekday group	PS‐matched weekend group	χ^2^ statistics	
Hospital‐acquired pneumonia	11 622 (3.2%)	3473 (3.1%)	1.4	0.23	3540 (3.2%)	3473 (3.1%)	0.7	0.42
Pulmonary embolism	15 627 (4.3%)	4744 (4.3%)	0.2	0.67	4712 (4.2%)	4744 (4.3%)	0.1	0.74
Myocardial infarction	325 (0.1%)	105 (0.1%)	0.2	0.62	112 (0.1%)	105 (0.1%)	0.2	0.63
Urinary tract infection	11 212 (3.1%)	3304 (3.0%)	3.5	0.06	3396 (3.0%)	3304 (3.0%)	1.3	0.25
Acute renal dysfunction	644 (0.2%)	185 (0.2%)	0.6	0.45	202 (0.2%)	185 (0.2%)	0.8	0.39
Dementia	5168 (1.4%)	1554 (1.4%)	0.3	0.57	1584 (1.4%)	1554 (1.4%)	0.3	0.59
Mortality during hospitalization	6448 (1.8%)	2061 (1.9%)	3.2	0.08	1929 (1.7%)	2061 (1.9%)	4.4	0.035

One‐to‐one PS matching was performed.

Age is shown as mean ± standard deviation; *P*‐values of <0.001 are considered significant by the Student's *t*‐test and χ^2^ test. PS, propensity score.

Although the mortality results during hospitalization were insignificant between the groups, the *P*‐value was low, so we conducted further investigation. Table 3 shows the multivariate logistic regression analysis outcomes to identify risk factors for mortality during hospitalization in patients with hip fractures. Among patients with hip fractures, several risk factors were identified as significantly associated with mortality during hospitalization: an increase in age by 1 year was associated with a ratio of 1.066 (95% CI: 1.061–1.071, *P* < 0.0001); male sex was associated with a ratio of 2.769 (95% CI: 2.590–2.961, *P* < 0.0001); diabetes was associated with a ratio of 1.214 (95% CI: 1.122–1.314); chronic renal dysfunction was associated with a ratio of 2.045 (95% CI: 1.847–2.264, *P* < 0.0001); and chronic lung disease was associated with a ratio of 2.437 (95% CI: 2.090–2.842, *P* < 0.0001). Weekend admission tended to be associated with the risk of mortality during hospitalization, with a ratio of 1.071 (95% CI: 1.005–1.140, *P* = 0.03).

**Table 3:** Multivariate logistic analysis of risk factors for mortality during hospitalization

Variable	Odds ratio (95% CI)	χ^2^ statistics	*P*‐value
Age	1.066 (1.061–1.071)	782.4	<0.0001
Sex (male)	2.769 (2.590–2.961)	826.5	<0.0001
Weekend admission	1.071 (1.005–1.140)	4.5	0.03
Hypertension	0.724 (0.676–0.775)	88.6	<0.0001
Diabetes	1.214 (1.122–1.314)	22.2	<0.0001
Cerebrovascular disease	1.052 (0.952–1.163)	0.97	0.32
Chronic renal dysfunction	2.045 (1.847–2.264)	161.9	<0.0001
Ischemic heart disease	1.043 (0.936–1.163)	0.6	0.45
Dementia	1.114 (1.034–1.200)	8.2	0.005
Chronic lung disease	2.437 (2.090–2.842)	104.1	<0.0001

*P*‐values of <0.001 are considered significant by the χ^2^ test. CI, confidence interval.

**Figure 2:**
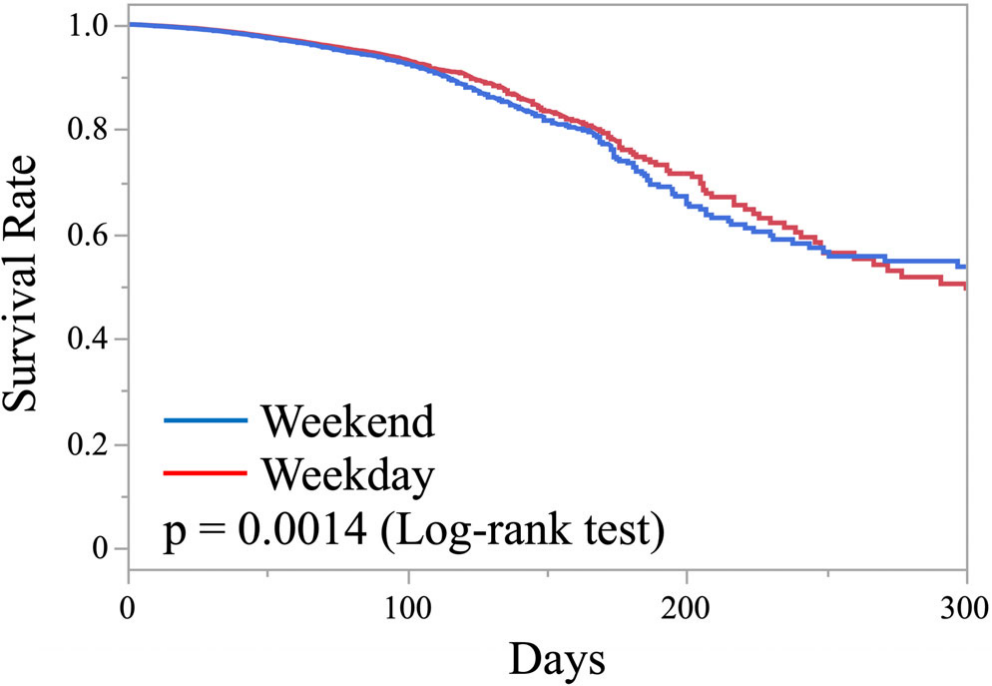
Survival rates of weekday and weekend admission groups. Results are expressed in the Kaplan–Meier curve. *P* = 0.0014 by log‐rank test.

Figure 2 shows the survival rates. The 30‐day survival rates for patients who continued to be hospitalized for the treatment of complications were 98.8% for the weekday admission group and 98.9% for the weekend admission group. The log‐rank test did not find a significant difference in survival rates between the groups over the entire period (*P* = 0.0014).

## Discussion

The objective of this study was to evaluate, through a comprehensive database of hip fractures in elderly Japanese patients, whether weekend hospitalization was correlated with the incidence of pneumonia, pulmonary embolism, and mortality during hospitalization. The study findings indicated that weekend admission for hip fractures was not associated with an increased risk of hospitalization‐related pneumonia, pulmonary embolism, myocardial infarction, urinary tract infection, acute renal failure, and dementia after adjusting for confounding factors in a multivariate analysis. However, the data suggested a potential association with elevated mortality rates. Overall, the effects of weekend admissions on patient outcomes appeared to be limited among the elderly Japanese.

Even in reports of the impact of weekend hospitalization on life expectancy, the increase in risk is small or somewhat ambiguous in setting the level of statistical significance, and the impact is relatively limited.^12^, ^24^ There are reports examining weekend hospitalization and surgical delays. Several studies have reported that weekend hospitalization is associated with delayed surgery and that this is related to the occurrence of sequelae and prognosis after surgery.^25^, ^26^, ^27^ On the other hand, studies that deny the effect of weekend hospitalization for hip fractures also report that delay in surgery has a negative impact on outcomes,^14^, ^15^ so it is still important to note that surgery should be performed within 48 h of injury to affect outcomes in elderly patients with hip fractures.^9^, ^10^, ^11^, ^19^, ^21^ A systematic review and meta‐analysis found that performing hip surgery within 48 h of admission significantly lowers mortality and reduces perioperative complications in elderly patients with acute hip fractures. The study did not find the day of the week as a significant risk factor.^28^

In the present study, the weekend hospitalization group had a larger percentage of patients who underwent surgery within 2 days of admission, indicating a shorter hospital stay. On the other hand, this study also noted that the use of blood transfusions during surgery was higher among patients hospitalized on weekends, suggesting that this group may face challenges related to preoperative physical conditioning and the surgical system's readiness. This observation implies that weekend admissions might be associated with more severe preoperative conditions or a less optimized surgical environment, potentially influencing patient outcomes. In contrast, the 30‐day survival rate was 98.8% for the weekday admission group and 98.9% for the weekend admission group, with good results for both groups. The results of this study suggest that Japanese hospitals participating in the DPC have established a system in which surgery can be performed within 2 days of admission, even for weekend admissions. Like in previous studies, chronic renal failure, diabetes, and dementia were associated with mortality risk in elderly hip fracture patients.^29^

This large study has several limitations, which are discussed below. First, the study population included hip fracture patients treated exclusively in acute care hospitals reported in the DPC data system. This does not include patients admitted to non‐DPC‐reported beds, which account for 30% of all general hospital beds, or patients who were never treated in an acute care hospital.^17^ Second, DPC‐based studies cannot assess the severity of a patient's symptoms. Third, one of the limitations of the study is that the use of anticoagulants was not ascertained. Finally, another limitation of the study is that the risk of death in the long term after discharge was not assessed; further large‐scale studies based on actual patient data are needed.

## Conclusion

In Europe and the United States, surgery within 48 h of injury has been reported to be important for good outcomes in elderly patients with hip fractures. On the other hand, in recent years, many reports have shown that the impact of weekend hospitalization in hip fracture patients is limited. A large DPC‐based study of Japanese patients also showed that weekend hospitalization was not associated with an increased risk of various sequelae and had no apparent association with an increased risk of mortality during hospitalization. This suggests that, even in cases of weekend admission, Japanese doctors may recognize the importance of performing surgery as soon as possible after hospitalization in the case of hip fractures in the elderly.

## Disclosure statement

The authors declare they have no conflict of interest regarding this study.

## Author Contributions

All authors are responsible for the work described in this paper. YM, KT, HT, NM, KFus, KFuj, and TA were involved in the study's conception, design, or planning. YM and HT were involved in the data analysis. YM, KT, HT, NM, KFus, KFuj, and TA interpreted the study results. All authors contributed to the critical review and approved the final manuscript.

## Data Availability

The data that support the findings of this study are available on request from the corresponding author. The data are not publicly available due to privacy or ethical restrictions.
